# GRANT Motif Regulates CENP-A Incorporation and Restricts RNA Polymerase II Accessibility at Centromere

**DOI:** 10.3390/genes13101697

**Published:** 2022-09-22

**Authors:** Hwei Ling Tan, Ee Sin Chen

**Affiliations:** 1 Department of Biochemistry, Yong Loo Lin School of Medicine, National University of Singapore, Singapore 117596, Singapore; 2 NUS Center for Cancer Research, Yong Loo Lin School of Medicine, National University of Singapore, Singapore 117599, Singapore; 3 National University Health System (NUHS), Singapore 119228, Singapore

**Keywords:** CENP-A, centromere, RNA polymerase II, N-terminus, histone, chromatin, epigenetic

## Abstract

Precise chromosome segregation is essential for maintaining genomic stability, and its proper execution centers on the centromere, a chromosomal locus that mounts the kinetochore complex to mediate attachment of chromosomes to the spindle microtubules. The location of the centromere is epigenetically determined by a centromere-specific histone H3 variant, CENP-A. Many human cancers exhibit overexpression of CENP-A, which correlates with occurrence of aneuploidy in these malignancies. Centromeric targeting of CENP-A depends on its histone fold, but recent studies showed that the N-terminal tail domain (NTD) also plays essential roles. Here, we investigated implications of NTD in conferring aneuploidy formation when CENP-A is overexpressed in fission yeast. A series of mutant genes progressively lacking one amino acid of the NTD have been constructed for overexpression in wild-type cells using the intermediate strength *nmt41* promoter. Constructs hosting disrupted GRANT (Genomic stability-Regulating site within CENP-A N-Terminus) motif in NTD results in growth retardation, aneuploidy, increased localization to the centromere, upregulated RNA polymerase II accessibility and transcriptional derepression of the repressive centromeric chromatin, suggesting that GRANT residues fine-tune centromeric CENP-A incorporation and restrict RNA polymerase II accessibility. This work highlighted the importance of CENP-A NTD, particularly the GRANT motif, in aneuploidy formation of overexpressed CENP-A in fission yeast.

## 1. Introduction

Proper segregation of chromosomes during cell division is essential for precise inheritance of the genomes and the loss of its fidelity often underlies human diseases, such as hereditary syndromes and cancers [1,2,3]. During M-phase, spindle microtubules emanate from the microtubule organization centers at the cell poles to capture the chromosomes at a locus termed centromere. The centromere hosts a specialized chromatin differentiated from the euchromatic and peri-centromeric heterochromatin by the enrichment of nucleosomes containing a centromere-specific histone H3 variant, CENP-A, in place of the canonical histone H3 [4].

Centromeric DNA of different organisms varies in sequences and length, yet all functional centromeres contain CENP-A-nucleosomes, which serve as the epigenetic determinant that specifies the position of centromeres within the chromosomes [5]. Indeed, in vitro reconstitution experiments demonstrated that the incorporation of CENP-A-nucleosome represents the critical functional step for the assembly of kinetochore [6]. CENP-A-nucleosomes erect the foundation to organize the constitutive centromere-associated network (CCAN) and the outer kinetochore KNL1-Mis12-Ndc80 (KMN) complex to serve as the adaptor for spindle microtubule attachment [7,8].

The expression level of CENP-A is critical for the maintenance of genomic stability. Depleting CENP-A levels in cells of organisms from yeasts to human results in delocalization of kinetochore components, compromised mitotic spindle attachment, chromosome missegregation and loss of cell viability [9,10,11,12,13]. Homozygous CENP-A null mice presents amorphic phenotypic manifestation characterized by loss of cell viability at 6.5 days post-conception [11]. Conversely, upregulating CENP-A levels leads to hypermorphic phenotypes associated with tumorigenesis in liver, lung, breast, bone, ovarian and colorectal cancers [14,15,16,17,18,19,20]. Overexpressed CENP-A in primary colorectal cancer cells was observed to mistarget to chromosomal arms and assemble into ectopic centromere-like structures, resulting in aneuploidy and tumor progression [19]. These observations raise interest for CENP-A overexpression to be a potential prognostic cancer biomarker [19,21,22,23,24].

The precise localization of CENP-A to the centromere depends on its interaction with a multitude of chaperones, which bind to different *cis*-acting regions within the CENP-A protein. CENP-A possesses a histone fold domain (HFD) that hosts a CENP-A targeting domain (CATD); an important Holliday junction recognition protein (HJURP) binding site for CENP-A incorporation into the nascent centromeric DNA [25]. Centromeric loading of fission yeast (*Schizosaccharomyces pombe*) CENP-A (SpCENP-A) is dependent also on Sim3—a human nuclear autoantigenic sperm protein (NASP)-like factor—proposed to hand over SpCENP-A to HJURP/Scm3 [26,27,28,29]. Sim3 binds a proline-rich genomic stability-regulating site within the CENP-A N-Terminus (GRANT) motif in a manner that depends on isomerization of the proline residues therein [29]. Recently, transcription of the centromeric DNA sequences is also implicated in centromeric targeting of CENP-A in various organisms, especially during the de novo incorporation of new CENP-A into newly replicated centromeric DNA. Factors that confer transcription regulation including RNA polymerase II (RNAPII), facilitating transcription (FACT) complexes and even a GATA-type transcription factor (Ams2) have been shown to impact centromeric localization of CENP-A [30,31,32,33,34].

In this work, we investigated the influence of NTD on cell proliferation and centromeric localization when SpCENP-A was overexpressed. Our results showed that overexpressed mutant CENP-A with incomplete or disrupted GRANT motif led to the over presence of CENP-A at centromere, chromosome missegregation, increased RNAPII accessibility and transcription at the core centromeric sequences, suggesting a safeguarding function of the CENP-A NTD through restricting RNAPII binding to core centromere in wild-type (WT) fission yeast cells.

## 2. Materials and Methods

### 2.1. Fission Yeast Manipulations

YEA, EMM and SPA media were prepared as previously reported [29,35,36,37]. The construction of SpCENP-A N-terminal truncation plasmids via recombinant DNA cloning in *Escherichia coli* and the transformation into fission yeast by the lithium acetate method for inducible expression were previously reported [36]. Mutant *cnp1* genes were cloned into REP41N plasmids carrying the inducible *nmt41* promoter with LEU2 selection marker [33] and transformed into WT fission yeast cells with *leu1* genetic background, which were maintained on EMM-leucine media. Fission yeast cells transformed with plasmids were grown overnight in 100 µM thiamine (Sigma-Aldrich, St Louis, MO, USA) to log phase and washed five times with autoclave-sterilized water to induce the *nmt41* promoter for 18 h.

### 2.2. Microscopy

To study nuclear phenotypes, cells were fixed for 15 min on ice with 1/10 (*v*/*v*) glutaraldehyde and washed thrice with ice cold 1 × PBS before mounting on slides with an equal volume of 100 µg/mL 4′,6-diamidino-2-phenylindole (DAPI) (Sigma-Aldrich) and observed using a Nikon Eclipse Ti-E inverted microscope using Plan-Apo 100× objective lens under oil immersion (Nikon, Tokyo, Japan). Image acquisition was performed with Nikon NIS-Elements AR software.

### 2.3. Serial Dilution Spotting Growth Assay

This assay was performed as previously reported [37,38,39,40]. Log phase cultures were amplified in EMM-leucine liquid media overnight with thiamine, which was removed by washing cells with water. The cells were constituted to OD_600nm_ = 0.5 (approximately 1 × 10^7^ cells/mL), serially diluted five times with EMM-leucine media. Then, 2.1 µL of each serially diluted cell suspension were spotted on EMM-leucine and EMM-leucine + 100 µM thiamine agar media. Growth was documented on day 3 and 7.

### 2.4. RNA Extraction and Reverse Transcriptase PCR Analysis

Cell pellets were homogenized in 200 µL TRIZOL (Thermo-Fisher Scientific, Waltham, MA, USA) and glass beads using FASTPREP-24 homogenizer (MP Biomedicals, Irvine, CA, USA). Chloroform (Sigma-Aldrich) was added to separate aqueous from organic phases. RNA in the aqueous phase was ethanol precipitated. Concentration of RNA obtained was determined with Nanodrop spectrophotometer (Thermo-Fisher Scientific). RNA was subjected to DNase I treatment at 37 °C for 3 h at a concentration of 1 U DNase I/µg RNA. Thereafter, the reaction mix was extracted twice with phenol:chloroform:isoamyl alcohol (25:24:1) (PCI) (Sigma-Aldrich) and ethanol precipitated. A total of 1 µg RNA was reverse transcribed to cDNA with QuantiTech reverse transcription kit (Qiagen, Hilden, Germany) according to the manufacturer’s instruction. The resultant cDNA was quantified by real-time PCR analysis. The forward and reverse PCR primers used to amplify the DNA within the centromeric core domain (Supplementary Figure S1) were 5′-TTACGCTTCACCTAGTTTCC-3′ and 5′-ATTATTTTCCAGTATGCTGATG-3′, respectively [41].

### 2.5. Chromatin Immunoprecipitation (ChIP)

ChIP was performed as previously reported [29,42,43]. Induced overnight cultures expressing SpCENP-A NT proteins were crosslinked with 3% (*w*/*v*) paraformaldehyde (Sigma-Aldrich) for 30 min on ice and then quenched with 2.5 M glycine. Cells were washed and resuspended in lysis buffer (50 mM HEPES (Sigma-Aldrich) pH 7.5, 100 mM NaCl (Sigma-Aldrich), 0.1% sodium deoxycholate (Sigma-Aldrich), 1 mM dithiothreitol (Sigma-Aldrich), 1 mM phenylmethylsulfonyl fluoride (Sigma-Aldrich), 1 × cOmplete protease inhibitor cocktail (Roche, Basel, Switzerland), homogenized with glass beads using FASTPREP-24 (MP Biomedicals), subjected to sonication using a Branson Sonifier at 80% duty cycle, output 1/1.1) twice with 30 s pulses and 5 min rest on ice in between pulses. After setting aside the whole cell extract (WCE), the extracts were mixed with 1.2 µg/sample primary antibody overnight. In total, 15 µL bed volume of protein G agarose per sample (Roche) was added to adsorb the antibody for washing. The ChIP samples were reversed crosslinked at 65 °C overnight, digested by proteinase K (Roche), PCI-extracted and ethanol-precipitated with 1 µg glycogen carrier. The ChIP and WCE DNAs were quantified using real-time PCR (Applied Biosystems, Waltham, MA, USA) with iTaq SybrGreen reagent (Bio-Rad, Hercules, CA, USA). Fold enrichment was derived from 2^−ΔΔCt^ calculations. The aforementioned primers [41] were employed for the amplification of ChIP DNA.

### 2.6. Statistical Tests

Statistical significance was denoted by *p* value < 0.05 (*) and <0.01 (**) using Student’s *t*-test. Statistical analyses and graphical output were obtained using Microsoft Office Excel (ver. 16.64, Redmond, WA, USA), and R (ver. 4.04, GUI 1.74, Open sourced from R-project).

## 3. Result

### 3.1. Overexpressed Truncated cnp1 Genes Result in Dominant Negative Growth Retardation

**Figure 1:**
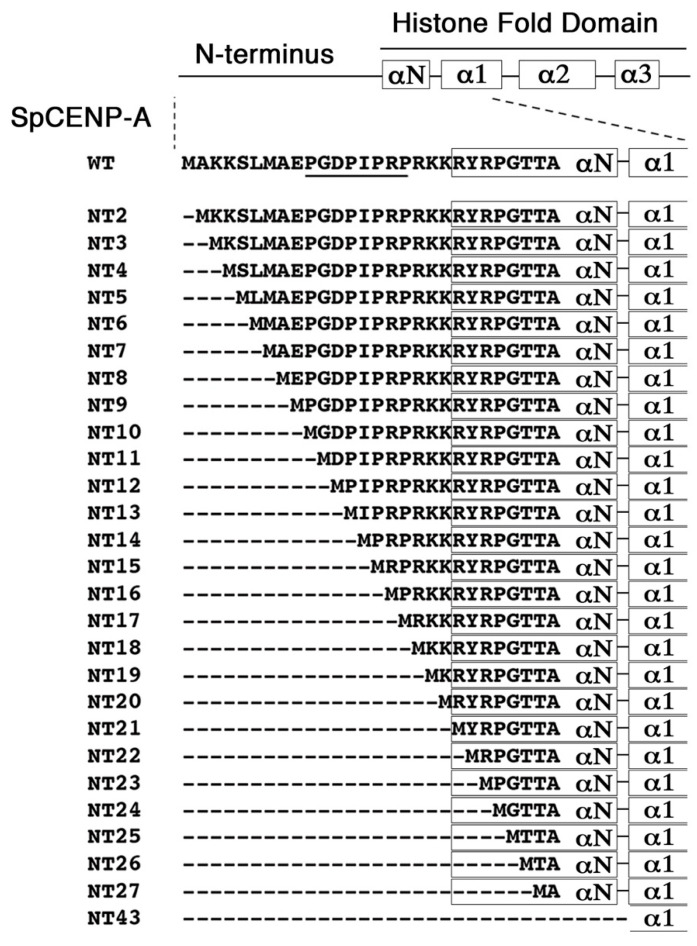
**Schematic diagram of *cnp1* NT mutant plasmids construction.** Systematic truncation of amino acid resides from the N-terminus of SpCENP-A was performed. NTx, NTD truncation with x denoting amino acid residue position. αN-α3, α helices. GRANT motif underlined.

To understand the influence of the N-terminal tail domain (NTD) on the cells overexpressing CENP-A, we systematically constructed a series of mutant *cnp1* genes expressing truncated NTD progressively losing one amino acids of the NTD (NT2 to NT20, NT: N-terminus truncated) and the α-N helix (NT21 to NT27), such that NT21 lost the entire NTD, while NT43 lost the NTD and the α-N helix. WT denotes the wild-type *cnp1^+^* gene that expressed the full length SpCENP-A protein (Figure 1). These 27 mutant and WT genes were cloned into REP41 plasmid to express them under the control of the inducible *nmt41* promoter [29,36]. Most of these truncated proteins (NT10 to NT43) were expressed at roughly the same level as the FL protein [36].

**Figure 2:**
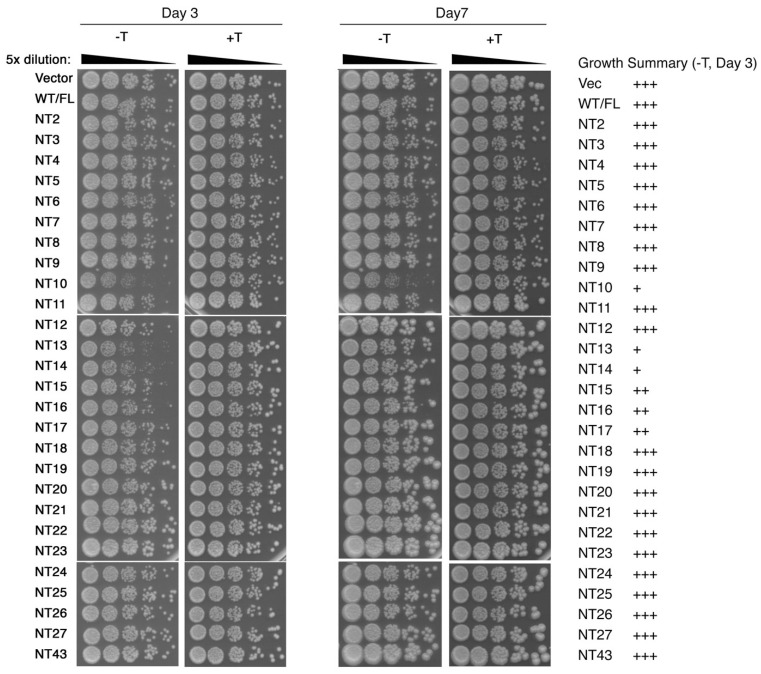
**Serial dilution growth assay of WT strain overexpressing cnp1 NT mutant plasmid constructs.** Growth was documented at day 3 (**left**) and 7 (**middle**) after serial dilution. The amount of growth of cells on day 3 was assessed visually and summarized (**right**), with ‘+++’ depicting growth comparable to WT transformed with empty vector (vec) and ‘++’ representing better growth than ‘+’. +/− T, with and without thiamine, respectively.

The effect of the overexpressed SpCENP-A NT mutant proteins (hereafter denoted as NTx, whereby the amino acid truncated is denoted by x) on the growth of WT cells were evaluated using serial dilution spot assay at permissive growth temperature of 30 °C, and compared to WT cells expressing the full-length *cnp1^+^* gene (WT) as well as with the empty vector. Cell growth was documented at an intermediate and stationary stages of growth at 3 and 7 days after cells were spotted on the plates (Figure 2). Cells expressing the WT SpCENP-A showed similar levels of growth relative to cells transformed with empty vector. However, growth retardation was observed in cells when NT10 to NT16 were induced by the removal of thiamine repressor (−T) as opposed to when the promoter was repressed (+T).

The slow growth phenotype was more apparent for NT10, NT13 and NT14 (approximately 25-fold), compared to NT11, NT12, NT15 and NT16 (approximately 5-fold) relative to the controls, and more apparent on day 3 than day 7. Among these, only NT10 showed slow growth on day 7 (Figure 2). Interestingly, these mutants disrupted the previously reported genomic stability-regulating site within the CENP-A N-terminus (GRANT) motif that stretches from residue proline-10 to proline-17, which we previously showed to regulate centromere-localization of CENP-A via the interaction with Sim3, a human NASP-like histone chaperone-like factor [29]. Cells expressing all other truncated versions of mutant SpCENP-A proteins besides NT10-16 showed a similar level of growth to the WT control (Figure 2).

### 3.2. Overexpressed GRANT-Truncated cnp1 Genes Disrupt Precise Chromosome Segregation

**Figure 3:**
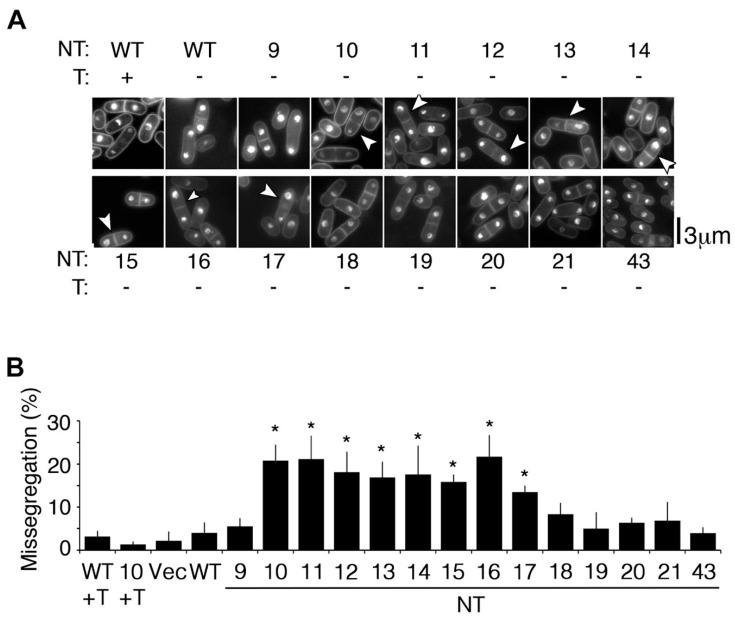
Chromosome missegregation phenotypes associated with ectopic expression of SpCENP-A NTD truncation mutant NT10-17 proteins. (**A**) Unequal chromosome segregation frequency (%) of WT cells expressing full-length (FL) and NTD-truncated (NT) mutants. Bar: 3 µm; +, −: with or without thiamine (T). Numbers 9–21, 43 indicate that of NT. (**B**) Quantification of the chromosome missegregation frequency of the WT strains ectopically expressed FL and NT proteins. Bar: standard deviation; *: *p* < 0.05 determined using Student’s *t*-test. Values were the mean of three independent replicates.

To investigate the reasons underlying the dominant negative growth retardation associated with the mutant *cnp1* genes that hosted truncation of the GRANT motif, we performed microscopic observations to quantify cell cycle-related phenotypes. The full-length (WT) and truncated *cnp1* genes (NT9 to NT21 and NT43) were expressed in log-phase WT cells, glutaraldehyde fixed for staining with DAPI, and compared with cells hosting empty vector as control (vec). Interestingly, unequal chromosome missegregation resulting in unequal-sized nuclei (Figure 3A) were reproducibly detected in approximately 20% mitotic and post-mitotic cells expressing NT10 to NT17 (Figure 3B). Although this level of missegregation is considerably lower than that observed in the conditional centromeric loss-of-function temperature-sensitive *cnp1-1* and *mis6-302* mutants, which exhibited ~60% and ~80% defects at 36 °C respectively [44,45], it is nevertheless comparable to the ~45% defects observed in *cnp1-1* cultured at semi-restrictive 33 °C [33].

Chromosome missegregation was not observed when NT10 expression was repressed by the addition of thiamine (Figure 3B, 10+T), indicating that overexpressed *cnp1* mutants led to chromosome segregation defects. Interestingly, cells expressing mutant *cnp1* genes truncated of residues before (NT9) and after the GRANT motif (NT18-21 and NT43) showed chromosome segregation similar to that of control cells expressing WT *cnp1^+^* gene and vec (Figure 3A,B). The results were observed in cells carrying a WT copy of *cnp1^+^* gene, thus indicating that a partial GRANT motif conferred detrimental dominant negative effects on the cells. Protein levels of the various *cnp1* genes expressed were shown to be comparable and the overexpression was estimated to be approximately 10-fold that of endogenously expressed SpCENP-A protein [29], showing that the chromosome missegregation phenotype does not arise from an aberrant expression peculiarity pertaining to NT10-17.

### 3.3. GRANT-Truncated Mutant SpCENP-A Derepressed Silencing at Inner Centromeric Chromatin

**Figure 4:**
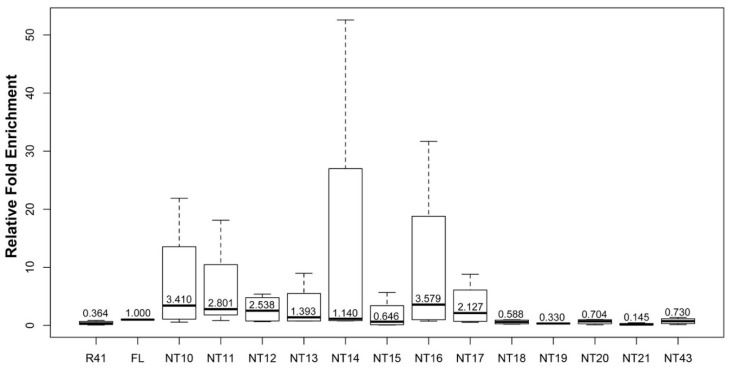
Upregulation in transcripts derived from centromeric core chromatin in strains expressing SpCENP-A NTD mutant proteins. Real-time RT-PCR detection of RNA transcripts from the centromeric central core domain DNA in strains expressing FL and NT10-21, NT43. Relative fold enrichment was normalized to FL. The strain transformed with empty Rep41 vector (R41) was employed as control. Bar: maximum and minimum, box: 25th and 75th percentiles. Bold lines in boxes: median. Graph from combined readings of at least two independent experimental replicates.

The transcriptionally silenced inner centromeric chromatin becomes de-repressed in mutants lacking SpCENP-A or SpCENP-A localizing factors and exhibit unequal chromosome segregation phenotypes [29,37,41]. We assessed the integrity of chromatin at the central centromeric core in cells expressing NT10-43 by determining the levels of transcript arose from the underlying centromeric DNA sequences. Coinciding with the high proportion of chromosome missegregation in NT10-17, high levels of transcript were observed to arise from the centromeric sequences (Figure 4). NT10, NT11, NT14 and NT16 appeared to show slightly higher levels of centromeric transcription, compared to NT12, NT13, NT15 and NT17, as revealed by real-time reverse-transcriptase PCR (RT-PCR).

**Figure 5:**
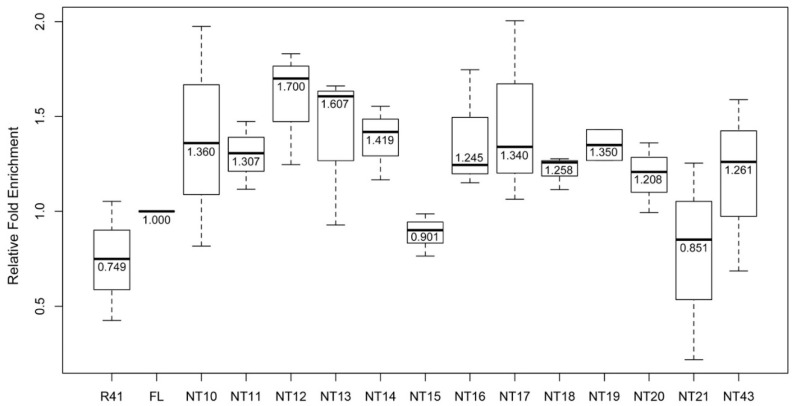
**Increased Rpb1 localization at the centromeric core chromatin in strains expressing SpCENP-A NTD truncation mutants.** Box-plots of relative fold enrichment from ChIP of Rpb1 in strains expressing FL and NT10-21, NT43. Relative fold enrichment was normalized to FL. The strain transformed with empty Rep41 vector (R41) was employed as control. Bar: maximum and minimum, box: 25th and 75th percentiles. Bold lines in boxes: median values. Graph from combined readings of at least two independent experimental replicates.

Next, we investigated the localization of RNA polymerase II (RNAPII) by performing ChIP on the largest subunit of RNAPII, Rpb1. We found a general increase in the localization of Rpb1 at the centromere in the presence of SpCENP-A N-terminal truncation mutants (Figure 5). With the exception of NT15, there is an increase in Rpb1 localization in strains expressing NT10 to NT20. Detectable levels of transcripts deriving from the centromeric sequences in cells overexpressing NT10-17 coincided with the increased presence of RNAPII at the centromere. Although a similar level of Rpb1 was detected in NT18-20 and NT43, we were unable to detect centromeric transcripts from these mutants (Figure 4).

### 3.4. Truncated Mutants Showed Higher Centromeric Localization Than Full-Length SpCENP-A

**Figure 6:**
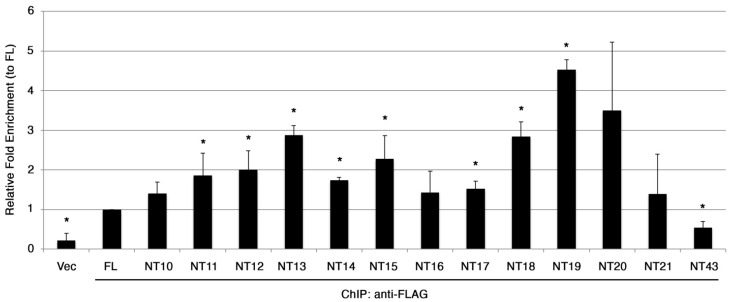
**Increased centromeric localization of SpCENP-A NTD truncation mutants.** Chromatin immunoprecipitation (ChIP) of FLAG-tagged WT and mutant SpCENP-A proteins at the centromeric core sequence. FL: full-length WT SpCENP-A; vec, vector, NT, N-terminal truncation. Relative fold enrichment was obtained by normalized to the FL. Bar; standard deviation, *: *p* < 0.05, determined by Student’s *t*-test. Values were the mean of five independent experiments.

Next, we assessed the localization of the truncated SpCENP-A proteins expressed from the mutant genes that were fused to an epitope FLAG tag, by performing chromatin immunoprecipitation (ChIP), relative to the binding of WT full-length protein at the inner centromeric core where SpCENP-A localizes [29,41]. Unexpectedly, we observed that most of the truncated proteins studied (NT10 to NT21) showed approximately 2–4-fold higher localization to the centromeric core sequences compared to FL. Although NT10, NT16, NT20 and NT21 also showed increased centromeric localization, the wide standard deviation probably precluded statistically significant difference from that of the FL. Binding of NT43 was significant decreased when compared to that of FL, indicating that loss of αN-helix abolished centromeric incorporation of SpCENP-A (Figure 6).

## 4. Discussion

Overexpression of CENP-A is associated with many cancers in humans. Here, we investigate the consequences of overexpressing CENP-A in fission yeast lacking part of or the entire NTD, leading to loss of centromeric integrity and chromosome segregation infidelity. Our results showed that the upregulated expression of full-length SpCENP-A induced from the intermediate strength *nmt41* promoter did not disrupt growth of the WT cells compared to that contained empty vector. However, the ectopic expression of mutant SpCENP-A proteins hosting disruption of the previously reported GRANT motif resulted in chromosome missegregation. This defect was associated with compromised centromeric chromatin integrity, indicated by the derepression of the silenced centromeric sequences and increased accessibility to RNAPII. Hence, moderate expression of SpCENP-A did not interfere with chromosome segregation unless the GRANT domain becomes disrupted. 

Expression of truncated SpCENP-A sequences proximal to the GRANT motif did not retard growth. However, in cells expressing mutant proteins with GRANT motif truncation—of even a single amino acid—disrupted growth retardation and chromosome missegregation were observed. Overproduction of the mutant protein with the GRANT motif fully truncated unexpectedly abolished the growth inhibitory effect. These results suggest that such dysfunctional phenotypes were not simply due to the loss-of-function arising from the disrupted NTD, otherwise expressing NT18, and beyond that, lacking the entire GRANT, would be expected to interfere with chromosome segregation as well. The presence of an incomplete GRANT, thus, acts in a dominant negative manner to interfere with chromosome segregation. Such dominant negative effect may be mediated via the interference with endogenous WT SpCENP-A function, either physically sequestering, or functionally occluding it from interacting with other centromeric factors, such as CENP-T [46,47] and Sim3 [29]. The presence of WT SpCENP-A at the centromeric core likely restrict the effect of the overexpressed protein, which may explain the milder chromosome missegregation phenotype relative to the total loss-of-function of SpCENP-A, for example in *cnp1-1* mutant at 36 °C [44]. Compromising the centromeric chromatin integrity would result in the loosening of centromeric chromatin compaction to permit RNAPII accessibility. Interestingly, upregulated transcripts of centromeric sequences can only be detected when NT10-17 was expressed but not NT18-43, suggesting that the intact GRANT motif may also repress transcription activity of RNAPII. 

Localization of mutants NT10-20 SpCENP-A were significantly upregulated at the centromere, suggesting that the region encompassing a.a. 10–20 is required for restricting centromere localization of SpCENP-A and prevent excessive incorporation of SpCENP-A into centromeric chromatin. However, the introduction of mutations into the GRANT sequences of the chromosomally expressed CENP-A protein resulted in loss-of-function phenotypes with attenuated centromeric localization due to the defects in the interaction of CENP-A with Sim3 chaperone. The dichotomy of GRANT regulating centromere targeting of SpCENP-A and concomitantly preventing excessive SpCENP-A incorporation could be mediated by cis-trans isomerization in a temporally regulated manner such that NTD may downregulate excessive centromeric incorporation of CENP-A during most of the cell cycle, while inducing timely regulated incorporation only during restricted window. Cis-trans isomerization of NTD may confer unique conformation to NTD to coordinate interaction with loading factors, such as the previously identified Sim3 [29]. Future research will be required to verify this hypothesis. 

CENP-A NTD may also regulate RNAPII occupancy at the transcriptionally repressive centromere, either directly or indirectly, to influence the compaction of the centromeric chromatin. In this aspect, our results suggest the CENP-A NTD can counteract RNAPII targeting to regulate its transcriptional activity at the centromere. This view is supported by the observation that even though RNAPII is localized to the centromere corresponding to the overexpression of truncated SpCENP-A beyond NT9, yet transcripts were only detected from cells expressing NT10-17, suggesting that GRANT may function to repress the activity of RNAPII. Such relationship of SpCENP-A with RNAPII may position NTD to exert a role in modulating RNAPII to incorporate SpCENP-A in a timely fashion, much like the intricacy of regulation of RNAPII on the outer heterochromatic centromere repeats, perhaps impacted by *cis*-*trans* prolyl isomerization of the GRANT motif [29,48,49,50].

The centromere localization of excessively expressed CENP-A in our experiments suggest that the centromeric chromatin is unexpectedly ‘spacious’, capable of still localizing much more SpCENP-A (NT19 showed approximately five-fold more ChIP signals relative to the FL, which itself has already been overexpressed [36]). This raises the possibility that the amount of CENP-A incorporated into the centromeric chromatin may underlie an important factor to fine-tune the role of CENP-A as a determinant of centromere formation and kinetochore assembly. Previous studies that quantified the level of CENP-A at centromeres of human retinal pigment epithelium cells revealed an extensive variation of CENP-A occupancy that is proportional to the protein expression levels, and the cells were able to survive with six-fold more of expressed CENP-A [51]. Other studies showed that centromeres of budding and fission yeast contain more CENP-A—four- and forty- fold more, respectively—than that estimated to be required for the formation of microtubule attachment sites [52]. We note that our results at the current state are insufficient to ascertain the ‘spaciousness’ of fission yeast centromeres. Future experiments will be required to assess nucleosome occupancy within the centromeric chromatin with respect to the precise locations of different types of SpCENP-A nucleosomes—those containing endogenously expressed SpCENP-A versus ectopically expressed full length and NT mutant SpCENP-A.

A regional centromere, which stretches for extended length and contains multiple centromeric nucleosomes, may allow the CENP-A nucleosomes to be distributed into the multiple disparate sub-compartments within the centromeric chromatin along with other centromeric proteins [53,54,55]. These compartmentalizations may constitute ‘extra space’ to accommodate a wide range of CENP-A levels. The formation of such modular architecture could attribute higher stability to the centromere in response to varying levels of protein expression of CENP-A (and also other centromere-binding proteins). This view is supported by observations that cells only start to missegregate chromosomes after a critical level of CENP-A has been lost from the centromere, which was about 50% of the total level of CENP-A in the human retinal pigment epithelium cells [51]. The capability of accepting more CENP-A may also be made possible by the folding of centromeric chromatin into a higher-order structure, which has been shown to be highly supercoiled [55,56,57,58,59]. These coiled domains can potentially be unwound to permit incorporation of more nucleosomes. As unwinding progressively occurs, it is expected that there would exist a critical threshold, beyond which would be expected to sufficiently disrupt the higher order structure within the centromere. This may be a possible underlying factor—at least in part—of the chromosome missegregation resulting from overexpressed SpCENP-A truncation mutants.

To assess whether the study conducted herein may bear translational implication in human diseases, particularly cancers, we conducted a search of cancer genome databases, but did not identify any mutation within human CENP-A NTD. More genome sequencing efforts in the future may uncover NTD mutations, particularly in the context of overexpression of CENP-A in cancer patients. However, we noted that several motifs within human CENP-A NTD—but not the histone fold domain—have been reported to be reactive sites targeted by autoimmune antibodies in systemic sclerosis [60,61]. Many of these motifs are proline-rich and also contain arginine residues, such as a KPxxPxR [60], to which GRANT (PDGPIPRP) highly resembles. Systemic sclerosis is a debilitating disease with hitherto ill-defined pathogenesis. The results reported herein raise the possibility that influence of proline-rich motifs within NTD of CENP-A on chromosome segregation may constitute a disease trigger, which can be addressed in future cellular or animal autoimmune disorder models.

## 5. Conclusions

Overexpressing CENP-A hosting truncated the GRANT motif, resulting in chromosome missegregation and upregulated CENP-A centromeric localization that were correlated with increased accessibility of RNA polymerase II and transcriptional derepression of the central core centromeric DNA sequences.

## Data Availability

Not Applicable.

## Supplementary Materials

The following supporting information can be downloaded at: https://www.mdpi.com/article/10.3390/genes13101697/s1, Figure S1: Supplementary Figure S1.
