# Supplementary figures and images for: GRANT Motif Regulates CENP-A Incorporation and Restricts RNA Polymerase II Accessibility at Centromere
Source: Genes (Basel). 2022 Sep 22;13(10):1697. doi: 10.3390/genes13101697 (PMC9602348; doi:10.3390/genes13101697)

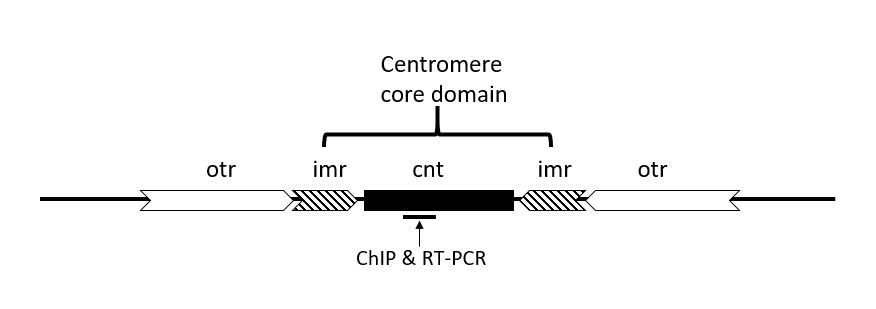

Supplement: Supplementary file 1 [file genes-13-01697-s001.zip › Supplementary Figure S1.JPG]
